# A Case of Hysterectomy for the Removal of an Enormously Large Fibroid Uterus

**Published:** 1895-07

**Authors:** J. H. Evans

**Affiliations:** Palestine, Texas


For the Texas Medical Journal.

### A CASE OF HYSTERECTOMY FOR THE REMOVAL OF AN ENORMOUSLY LARGE FIBROID UTERUS.

BY J. H. EVANS, M. D., PALESTINE, TEXAS.

EDITH S., a negress, aged 32 years; widowed, and mother of two children (both dead, having died in infancy), having always enjoyed good health and inherited a good family history as to physical strength. About six years ago, she first noticed an enlargement in the abdomen, which gradually grew, up the time she consulted me in November, 1894, in regard to her condition. She was at that time, flooding profusely, and suffering from uterine colic, and had been for some time, and could get no relief from the usual remedies prescribed in such cases. She had seen quite a number of physicians in regard to her condtition, and so far as I can learn, the majority of them were in favor of an operation, and so advised it; and being of that opinion myself, I explained to her the nature of the operation and the probable danger of its performance. She, after due consideration, consented to have it done. Having thoroughly prepared her in the usual way, and with the able assistance of Drs. Jameson, Hathcock, Dunn and Link, I proceeded as follows: Of course, the first thing we did was to thoroughly cleanse the field of operation and render our hands, instruments, etc., thoroughly aseptic. I then made an incision about six inches long, in the linea alba, and upon entering the peritoneal cavity (which was somewhat difficult owing to the excessive amount of fat), I found a tumor, about the size of a child's head, which proved to be an interstitial fibroid. The whole uterus was involved, and its walls were about the same thickness in all parts, about two inches, and of a stony hardness. The cavity of the uterus was in the center of the tumor, and not larger than an almond shell. The cervical canal was about four inches long, and about half the normal size. The tumor was movable; adhesions being exceedingly slight. I proceeded to tie and cut off each broad ligiment, which was easily done. I then passed a double silk ligature through the neck low down, tied and amputated. I then fastened the stump to the lower end of the incision, and treated it as an open wound. My main reason for carrying out the extra peritoneal mode of treatment was that the stump was unusually large, and I wished to avert the danger of secondary hemorrhage or septic infection. I approximated the edges of the peritoneum with animal ligatures and the skin with silk. The toilet of the operation was completed in the usual way, and with the exception of a few small stitch abscesses, my patient made an uninterrupted recovery, and is to-day, in the enjoyment of health and prosperity; weighing over 200 pounds. The only thing she fails to understand, and which worries her mind, is that her "flours" will not return (as she expresses it). I do not report this case because I claim originality in the mode of operation or treatment, but because it is an unusual operation in the interior, and because it would in my opinion be of interest to my brethren. I have not given greater details because I expect my readers to be learned in our profession and will readily supply such facts as are usual or as would necessarily occur.

